# Mapping the neural correlates of the effect of psycholinguistic variables on picture naming performance: a FDG-PET study across neurodegenerative diseases

**DOI:** 10.1186/s13195-025-01936-y

**Published:** 2025-12-20

**Authors:** Francesca Conca, Valentina Esposito, Cristina Polito, Gaia C. Santi, Daniela M. Gibbons, Silvia P. Caminiti, Cecilia Boccalini, Carmen Morinelli, Valentina Berti, Salvatore Mazzeo, Valentina Bessi, Alessandra Marcone, Sandro Iannaccone, Sandro Sorbi, Daniela Perani, Stefano F. Cappa, Eleonora Catricalà

**Affiliations:** 1 ICoN Cognitive Neuroscience Center, Institute for Advanced Studies, IUSS, Pavia, Italy; 2 Istituti Clinici Scientifici Maugeri IRCCS, Milan , Italy; 3 IRCCS Fondazione Don Carlo Gnocchi, Florence, Italy; 4 Istituti Clinici Scientifici Maugeri IRCCS, Bari , Italy; 5 Center for Mind/Brain Sciences (CIMeC), University of Trento, Rovereto, Italy; 6 Departmentof Brain and Behavioural Sciences, University of Pavia, Pavia, Italy; 7 Laboratory of Neuroimaging and Innovative Molecular Tracers (NIMTlab), Faculty of Medicine, Geneva University Neurocenter, University of Geneva, Geneva, Switzerland; 8 Research and Innovation Centre for Dementia-CRIDEM, Azienda Ospedaliero-Universitaria Careggi, Florence, Italy; 9 Department of Biomedical Experimental and Clinical Sciences, University of Florence, Florence, Italy; 10 Vita-Salute San Raffaele University, Milan, Italy; 11 IRCCS Policlinico San Donato, San Donato Milanese, Italy; 12 Department of Neuroscience, Psychology, Drug Research and Child Health, University of Florence, Florence, Italy; 13 IRCCS San Raffaele Hospital, Milan, Italy; 14 Department of Rehabilitation and Functional Recovery, San Raffaele Hospital, Milan, Italy; 15 IRCCS Istituto Auxologico, Milan, Italy

**Keywords:** Picture naming, Psycholinguistic variables, Neurodegenerative diseases, FDG-PET imaging

## Abstract

**Background:**

Picture naming performance is influenced by the properties of the stimuli and of the words to be retrieved, such as word length and lexical frequency. Significant inconsistencies, however, remain regarding the brain regions mediating these effects in neurodegenerative patients. In the present study, we addressed this issue by correlating regional cerebral metabolism with several naming-related variables in a large cohort of neurodegenerative patients, who are likely to exhibit naming impairments due to different mechanisms of cognitive dysfunction.

**Methods:**

A total of 178 patients classified within the Frontotemporal (FTD) and Alzheimer’s Disease (AD) spectra were administered a picture naming test validated for the Italian language (CaGi) and underwent a FDG-PET scan. Principal Component Analysis on 10 psycholinguistic variables resulted in the extraction of four components, labelled as word-form, visual, lexical, and semantic, according to the variables populating each of them. Using an item-level approach, the influence of each component on patients' performance was assessed and correlated with brain metabolism data from 11 left hemispheric Regions of Interest.

**Results:**

A simple word form and lexical structure were associated with better naming performance. The imaging findings reveal a distributed neural network, with fusiform gyrus supporting both visual and semantic features. Inferior frontal and posterior temporal/parietal gyri represented an interface between lexico-semantic and phonological properties. The anterior temporal lobe contributed to all the stages of picture naming. The two dementia spectra activated different areas in response to the same variables, in particular for the visual and semantic components, suggesting the presence of disease-specific compensatory mechanisms.

**Conclusions:**

Our results suggest a distributed neural network showing both commonalities and specificities in how picture and word properties influence naming performance. The network also seems capable of compensatory changes in the face of the extension of neurodegenerative processes.

**Supplementary Information:**

The online version contains supplementary material available at 10.1186/s13195-025-01936-y.

## Background

Picture naming is one of the most commonly used tests to assess language impairment in neurological patients. It entails a multi-component process, depending on a series of dissociable yet interacting stages, involving, at least, the mapping from visual input to semantic representation and the subsequent linkage to the word form for spoken output [42]. According to some proposals, the lexical access stage (i.e., mapping from semantics to phonology) is further partitioned into two steps, namely the lexical representation corresponding to the concept (also known as lemma, [59]) and the phonological retrieval stage [53]. Importantly, a deficit in any one of these components can result in naming errors.

Several variables related to the pictures and to their names have been shown to affect naming speed and/or accuracy in healthy subjects (see [83] for a meta-analysis), as well as in neurological conditions, particularly in post-stroke patients. The performance in picture naming tasks is facilitated by high lexical frequency, familiarity and typicality, short length, acquisition early in life, high phonological neighbourhood density and high number of distant semantic neighbours [3, 6, 26, 27, 43, 50, 57, 60, 72, 76, 80, 12, 67, 92, 112, 113], for exceptions see the absence of frequency and familiarity effects in the study by [77], or of length effects in [7].

The effect of these variables has been proposed to operate either on a specific and unique stage of the naming process, or on several/all stages [3, 33, 61]. In addition, the variables may interact with each other, making it difficult to disentangle their effects [41].

Studies investigating the brain regions affected by each variable in picture naming are scarce and heterogeneous in terms of population and variables included, and are difficult to compare (see Table 1 in the Supplementary Materials). The available findings have been collected in healthy participants, stroke patients and in carriers of small temporopolar epileptogenic lesions. The studies took into account from 1 to 8 variables, which were used independently in the statistical analyses or with a dimensionality reduction approach. The most frequently studied variables are frequency and word length, followed by familiarity and Age of Acquisition (AoA). Frequency involved posterior temporal areas and the inferior frontal gyrus [3, 39, 48], but see [115] for the lack of this effect), and has been suggested to contribute to phonological retrieval and encoding. Word length involved the superior temporal cortices [79, 115] and frontal areas, i.e. premotor cortex [79], supplementary motor and mid-frontal areas [115], suggesting a contribution to later processing stages, from phonological encoding to articulation. Familiarity and AoA shared an involvement of occipital areas, fusiform gyri [34, 48, 115, 117], and temporal pole [3, 14, 34, 107], suggesting a similar role in visual object recognition and semantic processing. Other variables have been less frequently investigated (see Table 1 in the Supplementary Materials).

**Table 1:** Demographic information for the whole sample, AD spectrum (i.e. typical AD, lvPPA and PCA), FTD spectrum (i.e. nfvPPA, svPPA, bvFTD, CBS, PSP), and for each patient group

	**Sex (M/F)**	**Age, years (mean ± sd)**	**Education, years (mean ± sd)**	**MMSE (mean ± sd)**	**Disease duration, months (mean ± sd)**
bvFTD, *n* = 16	8/8	71.06 ± 6.43	8.63 ± 4.40	24.0 ± 4.55	25.50 ± 8.46
nfvPPA, *n* = 8	6/2	70.88 ± 6.15	10.63 ± 6.19	22.94 ± 3.02	26.14 ± 18.15
svPPA, *n* = 25	10/15	67.28 ± 8.63	10.80 ± 3.72	22.27 ± 5.50	25.80 ± 15.87
CBS, *n* = 8	3/5	68.13 ± 7.29	12.88 ± 4.97	20.88 ± 3.62	20.38 ± 5.80
PSP, *n* = 13	6/7	69.00 ± 7.37	11.62 ± 4.01	25.35 ± 3.86	32.00 ± 16.43
FTD spectrum, *n* = 70	33/37	68.97 ± 7.50	10.67 ± 4.46	23.15 ± 4.67	26.3 ± 13.98
AD, *n* = 28	15/13	66.61 ± 9.67	9.39 ± 4.67	19.66 ± 4.49	27.43 ± 18.44
lvPPA, *n* = 47	28/19	69.49 ± 6.74	10.87 ± 4.50	21.55 ± 4.95	23.85 ± 10.55
PCA, *n* = 7	1/6	62.14 ± 8.39	12.71 ± 3.40	19.62 ± 3.86	23.07 ± 19.87
AD spectrum, *n* = 82	44/38	67.88 ± 8.19	10.52 ± 4.54	20.74 ± 4.75	25.02 ± 14.49
mixed PPA, *n* = 13	6/7	70.23 ± 6.51	9.92 ± 4.17	19.71 ± 7.09	25.62 ± 17.52
DLB, *n* = 13	10/3	70.00 ± 6.78	8.23 ± 3.94	21.70 ± 4.48	25.23 ± 14.21
Whole sample, *n* = 178	92/85	68.63 ± 7.69	10.37 ± 4.45	21.68 ± 5.02	25.58 ± 14.39

*AD* Alzheimer’s disease, *bvFTD* behavioral variant of frontotemporal dementia, *CBS* corticobasal syndrome, *DLB* dementia with Lewy bodies, *FTD* frontotemporal dementia, *lvPPA* logopenic variant of primary progressive aphasia, *MMSE* Mini-Mental State Examination, *nfvPPA* nonfluent variant of primary progressive aphasia, *PCA* posterior cortical atrophy, *PPA* primary progressive aphasia, *PSP* progressive supranuclear palsy, *sd* standard deviation, *svPPA* semantic variant of primary progressive aphasia

In this context, the available evidence in neurodegenerative diseases only derives from behavioral studies. A recent study suggested an effect of AoA and word length on the overall naming accuracy in a sample of patients with Primary Progressive Aphasia (PPA) including semantic, logopenic, non-fluent, as well as mixed cases [85]. A contribution of AoA on naming accuracy has been reported in additional studies of the semantic, logopenic [55], and non-fluent [106] variants. Naming accuracy in sv-PPA is also predicted by familiarity, frequency, and typicality [5, 89, 116]. In patients with Alzheimer’s Disease, naming accuracy is influenced by visual complexity, familiarity, frequency, AoA, and name agreement [37, 91, 99].

To the best of our knowledge, no evidence compared the neural correlates of the impact of multiple naming related variables in Frontotemporal (FTD) and Alzheimer’s Disease (AD) spectra. As an impaired performance in picture naming is frequently observed in patients affected by neurodegenerative diseases, studies in these patient populations may add relevant insights on the functional organization of picture naming. In particular, we may assess the role of brain regions not constrained by the vascular distribution territories, as in case of ischemic stroke.

In the present study, by using FDG-PET imaging, we aimed to explore the neural correlates of the effect of 10 psycholinguistic variables on picture naming accuracy in the AD and FTD spectra. To this purpose, we recruited a large group of neurodegenerative patients (*n* = 178) with a naming impairment possibly reflecting the involvement of different stages of the naming process.

## Materials and methods

### Participants

One hundred and seventy-eight Italian-speaking patients with a clinical diagnosis of neurodegenerative dementia were retrospectively collected from the databases of the Careggi University Hospital of Florence (100 patients) and of the San Raffaele Hospital of Milan (78 patients). Clinical diagnoses were formulated by expert neurologists (DP, AM, SI, SFC, SM, VB, SS) according to current criteria [4, 25, 45, 65, 69, 70, 90] based on clinical data, a comprehensive neuropsychological assessment and, when available, biomarker information.

Seventy patients belonged to the clinical spectrum of frontotemporal dementia (FTD), including behavioural variant of frontotemporal dementia (bvFTD) (*n* = 16) [90], semantic (svPPA) (*n* = 25) and non-fluent (nfvPPA) (*n* = 8) variants of primary progressive aphasia [45], progressive supranuclear palsy (PSP) (*n* = 13) [65], and corticobasal syndrome (CBS) (*n* = 8) [4]. We included 82 patients belonging to the clinical spectrum of Alzheimer’s Disease (AD), including the typical AD presentation (*n* = 28) [70], logopenic variant of PPA (lvPPA) (*n* = 47) [45], and posterior cortical atrophy (PCA) (*n* = 7) [25]. In the whole group analyses, thirteen patients with a clinical diagnosis of Dementia with Lewy Bodies (DLB) [69] were also included, as well as 13 patients with a diagnosis of mixed PPA (i.e. 3 lv/nfvPPA, 6 lv/svPPA, 1 nfv/svPPA, 3 mixed PPA unspecified).

AD and FTD spectra, as well as the single patient groups did not differ for age, years of education, sex, and disease duration (all p values at least > 0.164). On the Mini Mental State Examination (MMSE, corrected score), AD spectrum patients performed worse than those belonging to the FTD spectrum (t = −3.138, *p* = 0.002). See Table 1 for demographic and clinical data of the patients.

The study was conducted in compliance with the Helsinki Declaration and was approved by the local ethics committees. All participants gave informed consent to participate.

### CaGi picture naming test

All patients had been administered the picture naming test of the CaGi battery [19] based on 48 coloured photographs, which is widely adopted in Italian clinical and research contexts [17, 18, 20, 21, 85]. They were asked to orally name each visually presented stimulus, taking all the time they needed to respond. The response was counted as incorrect in case of visual, semantic, anomic, or phonological errors, as well as mixed errors and perseverations, in agreement with our previous work [21]. Articulatory distortions and other errors attributable to motor speech impairments were not classified as errors, provided that the response was intelligible, following the scoring procedures of the CaGi naming test [19]. No instances of unintelligible responses were observed in our sample. The differentiation between phonological and articulatory errors was based on the clinical judgment of two expert speech neuropsychologists (VE, CP), with no cases of disagreement.

For each stimulus, values of 10 psycholinguistic and semantic properties were considered, specifically: number of letters and syllables, phonological neighbourhood, visual complexity, visual relevance, frequency, familiarity, age of acquisition, concept relevance and semantic distance from centroid. All variables were derived from published norms for the Italian language; Table 2 below provides the complete list of variables, their descriptions and the references of the norms used.

**Table 2:** Psycholinguistic and semantic properties of the items of the CaGi naming test

Variable	Definition	Reference
number of syllables		[46]
number of letters		[46]
phonological neighbourhood	number words that can be constructed by changing a single phoneme of the target word	[46]
visual complexity	amount of details and intricacy of lines and edges of each picture	[110]
visual relevance	obtained by a nonlinear combination of dominance° and distinctiveness* of the visual features, calculated from the data reported by [19]	[19]
frequency (FREQ)	how often the word is used in written text, expressed as log-transform of 1 + number of occurrences over one million	[30]
familiarity (FAM)	how familiar the concept is on a scale ranging from 1 to 7, from extremely unfamiliar to extremely familiar	[19]
Age of Acquisition (AoA)	how early in life the concept was acquired, expressed on a 9-points scale with 1 indicating 2 years or younger and 9 indicating 13 years or older	[30]
concept relevance	nonlinear combination of dominance° and distinctiveness*, calculated from the data reported by [19]	[95]
semantic distance from centroid	distance between the concept and the centroid of the respective semantic category, namely a vector corresponding to the average of the vectors for the exemplars from that category, calculated from the data reported by [19]	[118]

°dominance = the number of participants who listed a specific feature for a specific concept; *distinctiveness = the number of concepts for which the semantic feature appears, divided by the total number of concepts in the database

### Analysis of behavioural data

Data analysis was performed using IBM SPSS Statistics (version 21) software.

#### Patients’ behavioural performance

The naming accuracy of each patient was classified as impaired or spared according to the normative data, and the percentage of impaired cases was calculated in the whole sample, in the AD (i.e. typical AD, lvPPA and PCA), and in the FTD spectrum (i.e. nfvPPA, svPPA, bvFTD, CBS, PSP).

#### Psycholinguistic variables

Before assessing the core organization of the ten variables of the CaGi stimuli, we assessed their collinearity with Pearson’s correlation analysis (2-tailed). In cases where the correlations exceeded |0.8|, one of the corresponding variables was excluded from further analysis. We then applied a dimensionality reduction approach using Principal Component Analysis (PCA) to condense the set of variables into a smaller set of components. Components with an eigenvalue > 1 were extracted and varimax rotated. This orthogonal rotation enhances the interpretability of the outlined components as it maximises the loading of a single variable into one component. To evaluate whether the data were suitable for PCA, we examined sample size adequacy using the Kaiser–Meyer–Olkin (KMO) measure and assessed the strength of inter-variable correlations using Bartlett’s test of sphericity.

#### Accuracy

Patients’ performance was calculated at the item level as the proportion of individuals who correctly named each item out of the total number of patients in the whole sample (/178) and out of the number of patients in each spectrum (/70 for FTD spectrum and/82 for AD spectrum). By item Pearson correlation analyses (two-tailed) were then adopted to explore the relation between patients’ performance and psycholinguistic variables, in term of PCA components. Bonferroni-Hochberg correction for multiple comparisons was adopted [71].

#### Weights

**Fig. 1:**
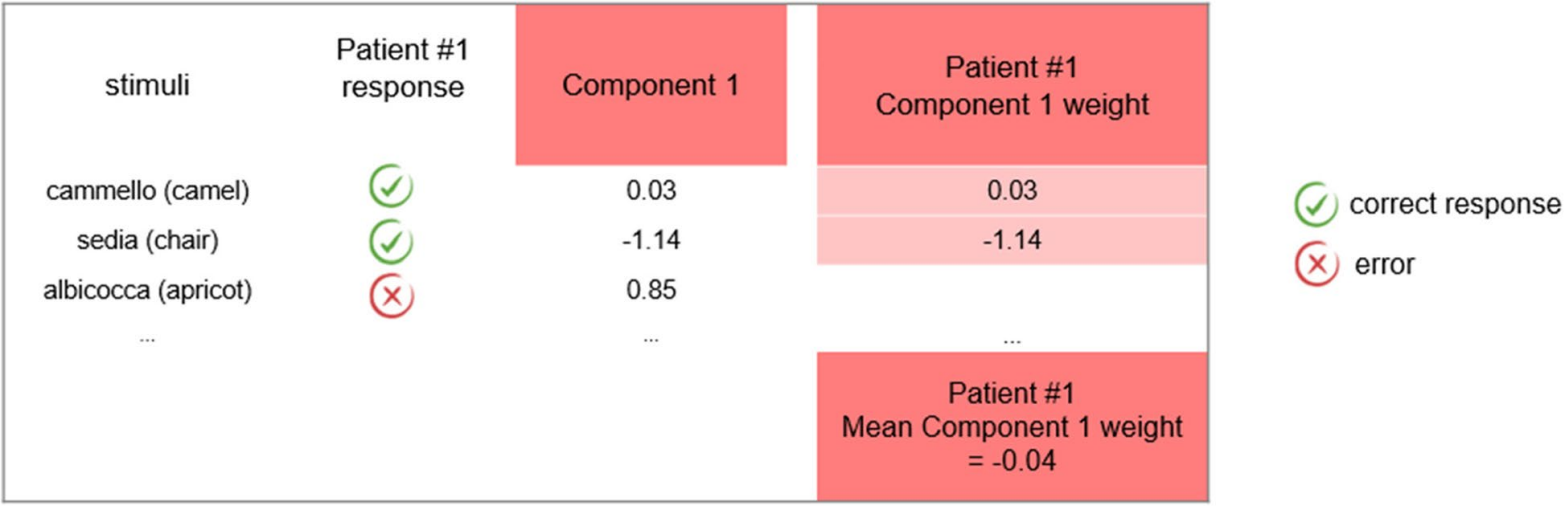
Schematic representation of the procedure adopted to calculate the weight of each PCA component for each patient. The figure includes examples for one component (Component 1- word form)

Following previous works [31, 35] a different difficulty score (weight) was assigned to each stimulus, following the assumption that items with higher values of frequency, familiarity etc. are easier to be named. Correctly naming more difficult items indicates a preserved status of semantic memory. Here, the difficulty of the items was operationalized on the basis of the PCA components involving the psycholinguistic variables, not in terms of individual variables. Each of the 48 items of the CaGi naming test was associated to a score for each PCA component, extracted as regression values from the PCA results. To extrapolate the weight of each PCA component on naming performance for individual cases, we considered only the items which were correctly named by each patient. By averaging the values derived from each component, we obtained a series of component weights for each patient. See Fig. 1 for a schematic representation of the procedure [31, 35].

### Imaging methods

#### FDG-PET acquisition

All patients underwent a FDG-PET scan acquisition, performed within three months from neuropsychological assessment according to the European Association of Nuclear Medicine (EANM) guidelines [109]. Before radiopharmaceutical injection, participants were fasted for at least six hours in order to ascertain that the blood glucose level was < 120 mg/dl. All subjects were under resting conditions with eyes closed, lying comfortably in a quiet room. The conventional scan was started 30–45 min after injecting [^18^F]FDG via a venous cannula, and continued for a total of 20 min. Patients were scanned with General Electric’s Medical Systems Discovery-STE scanner in Milan and Gemini TF PET/CT (Philips Medical Systems) in Florence. A multi-centre data collection approach has been previously employed in our studies [21, 22, 85, 94], based on the evidence that the optimized FDG-PET procedures are not influenced by the variability of the scanners, and represent a valid method for longitudinal and multi-centre studies [86]. Images were analysed using the single-subject procedure implemented in Statistical Parametric Mapping (SPM): 1) spatial normalization using a dementia-specific template [82],2 smoothing (isotropic 3D Gaussian kernel with a FWHM of 8 mm in each direction; and 3 global mean scaling for intensity-normalization (SUVr). Each patient’s scan was evaluated for hypometabolism using a validated SPM procedure [82], encompassing the comparison with a sample of healthy controls, as previously employed by our group [94]. SUVr were then extracted from 11 Regions of Interest.

For each spectrum (FTD and AD), we created a whole-brain map of hypometabolism to highlight the most affected brain areas in the two groups, with a significance level of *p* < 0.05 after applying a familywise error (FWE) correction for multiple comparisons, see Supplementary Materials.

#### Regions of Interest (ROIs)

Eleven left-lateralized Regions of Interest (ROIs) were considered, taking into account existing literature on picture naming tasks in patients and healthy subjects [3, 21, 29, 34, 39, 48, 74, 79, 84, 85, 107, 115, 117], see Table 3 in the Supplementary Materials. They included the middle occipital gyrus, posterior superior (pSTG) and middle temporal (pMTG) gyri, posterior fusiform (pFUS), middle temporal pole, anterior inferior temporal gyrus (aITG), inferior frontal gyrus, i.e. subdivided in pars orbitalis (IFGorb), triangularis (IFGtri) and opercularis (IFGoper), supramarginal gyrus and inferior parietal cortex. ROIs were derived from the Automated Anatomical Labelling (AAL) atlas [105], as in previous studies by our group [21, 22, 85, 94]. Partitions into anterior/posterior sections for fusiform gyrus and superior and middle temporal gyrus were defined based on the boundaries in the AAL atlas and according to Visser et al. [111]. Specifically, the MNI coordinates for posterior sections were Y =—19 to – 51 for superior/middle temporal gyrus and Y = −24 to −60 for fusiform gyrus. Note that our analysis focused exclusively on left-hemispheric regions due to an imbalance in hemispheric involvement. Specifically, the left hemisphere was well represented by the topographical profile of the patients in our sample, in particular in PPA patients, accounting for more than 50% of cases. Consequently, the right hemisphere may have had limited sensitivity in detecting effects related to the psycholinguistic variables under investigation.

**Table 3:** Schematic description of the analyses we performed with accuracy and weights

Aim	Metric	Level	Measure	Used for
to assess which psycholinguistic properties influenced picture naming performance	Accuracy	Item level	proportion of individuals who correctly named each item out of the total number of patients	correlation with the psycholinguistic variables, in term of PCA components
to assess how regional metabolism is associated to the ability to name items, which are characterized by specific psycholinguistic properties	Weights	Subject level	mean values of the PCA components of the correctly named items; for each component, one weight is calculated for each subject	correlation with the brain metabolism extracted from a priori defined ROIs

#### Correlation analyses

Correlation analyses (two-tailed) were used to explore the relation between, respectively, the accuracy (number of items named correctly by each patient) and the component weights (see the corresponding section above) and the metabolic values extracted from each ROI, adopting Bonferroni-Hochberg correction for multiple comparisons [71]. The analyses were performed in the whole sample and separately in the two spectra.

See Table 3 below for a schematic description of the analyses we performed.

## Results

### Behavioural results

#### Patients’ behavioural performance

The percentage of cases showing a pathological performance in the picture naming task in the whole sample was 45.51%, 55.71% in the FTD spectrum and 47.56% in the AD spectrum.

#### Psycholinguistic variables

**Fig. 2:**
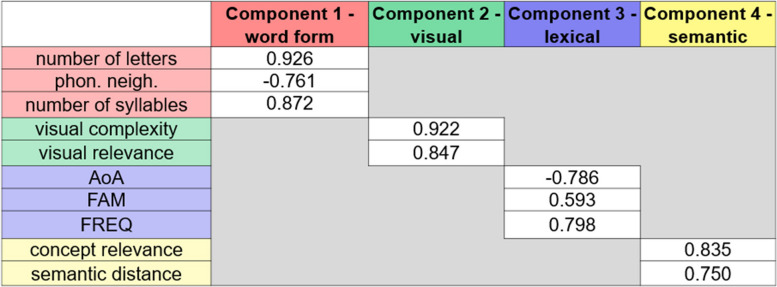
Component loadings of each variable onto the four extracted components; AoA = age of acquisition; FAM = familiarity; FREQ = frequency; phon. neigh. = phonological neighbourhood

Correlations among psycholinguistic variables are reported in the Supplementary Materials. No correlation exceeded |0.8| and all the variables were retained for the subsequent analyses. By applying PCA on the ten variables, four latent underlying components were extracted, accounting for 76.71% of the variance. Sample size was acceptable (KMO = 0.600), and correlations were sufficiently large (Bartlett's test = 179.796, *p* < 0.001). To aid the interpretations of the components, only loadings >|.5| are reported in Fig. 2. Component 1 (Word Form component) accounted for 31.04% of variance and was characterized by positive loadings of number of letters and syllables and negative loadings of phonological neighbourhood. High scores in this component indicated difficult words in terms of word form, with more letters and syllables and a few phonological neighbours. Visual complexity and visual relevance were grouped together in Component 2 (Visual component) accounting for 21.88% of variance. High scores in this component indicated visually difficult items, namely items showing high values of visual complexity and visual relevance. Component 3 (Lexical component), accounting for 13.40% of variance, was characterized by negative loadings of AoA and positive loadings of both FREQ and FAM. In other words, high scores in this component indicated easy concepts, i.e., frequent, familiar, and acquired early in life. Finally, Component 4 (Semantic component) accounted for 10.39% of variance and was characterized by positive loadings of both concept relevance and semantic distance from the centroid. High scores on this component indicated semantically easy concepts.

Item difficulty was thus operationalised by 4 different aspects, i.e. the 4 components of the PCA, namely word form, visual, lexical, and semantic. In particular, the most difficult items were characterised by high values in the word form and visual components, and low values in the lexical and semantic components.

#### Accuracy

Correlations between patients’ performance, considering the whole sample, AD and FTD spectra, and the 4 PCA components are reported in Table 4. Word form and lexical component correlated negatively and positively, respectively, with the proportion of correct responses in the whole sample and in the two spectra. Stimuli with simpler word form and lexical properties were more often named correctly.

**Table 4:** Results of the correlation analyses between the proportions of correctly named items and the 4 components, i.e., word form, visual, lexical and semantic in the whole sample, and in the AD and FTD spectra respectively

	**Word form**	**Visual**	**Lexical**	**Semantic**
**Whole sample,** r (*p*-value)	**-.381 (.008)**	-.040 (.788)	**.613 (<.001)**	.214 (.143)
**AD spectrum,** r (*p*-value)	**-.394 (.006)**	-.029 (.845)	**.605 (<.001)**	.223 (.127)
**FTD spectrum,** r (*p*-value)	**-.324 (.025)**	-.057 (.701)	**.630 (<.001)**	.186 (.206)

*r* = Pearson correlation coefficient. Significant, Bonferroni-Hochberg corrected results are in bold

### Imaging results

**Fig. 3:**
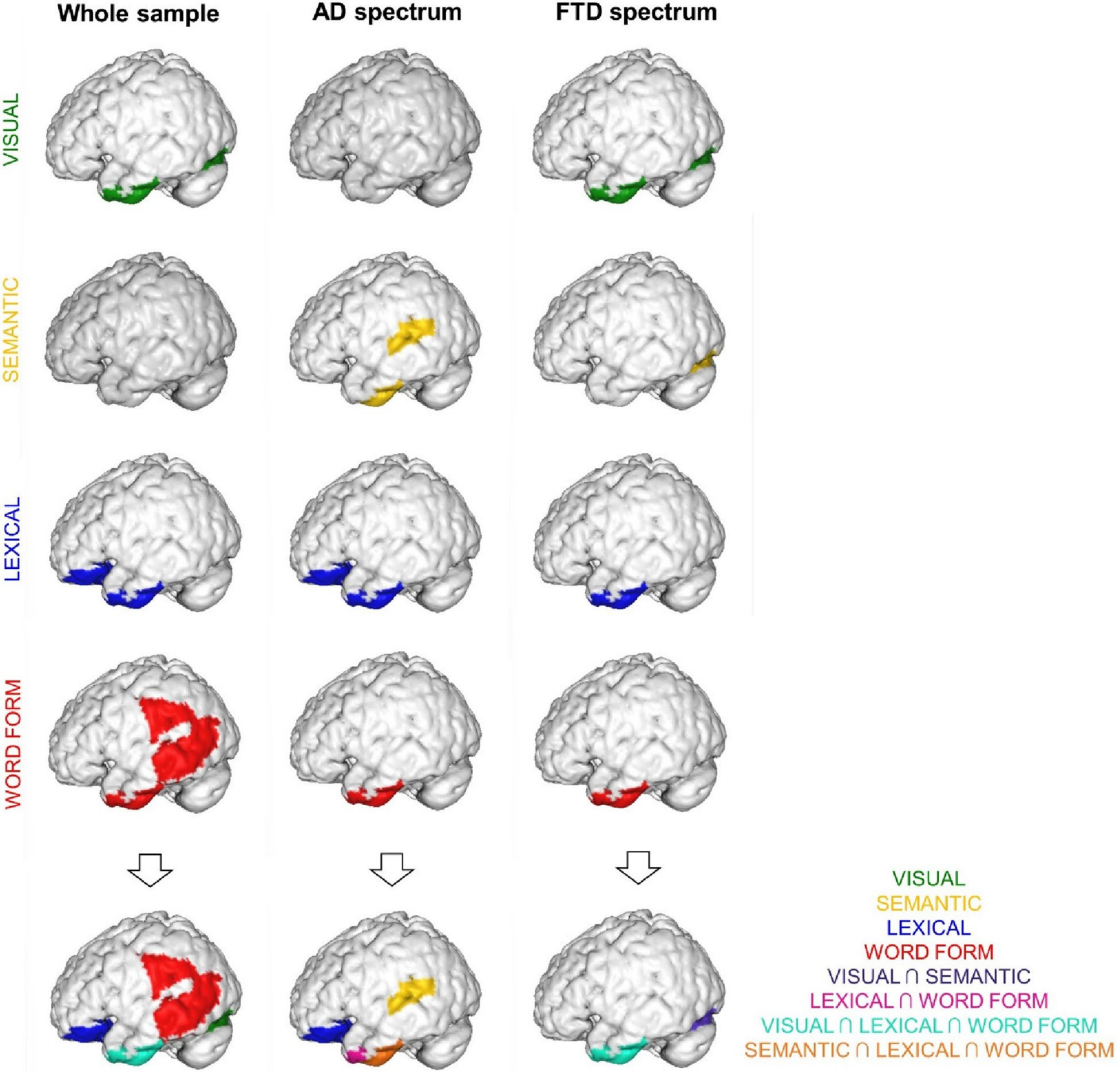
Anatomical rendering of the brain imaging results, obtained from the correlations between correct responses and the component weights (i.e. visual, semantic, lexical, and word form in row 1 to 5, and summation of the component weights in row 6) in the whole sample, AD and FTD spectra; ∩ indicates that a region correlates with the listed component weights

See Table 5 and Fig. 3 for the results of the correlation analyses between the component weights and the metabolism in the 11 ROIs, respectively in the whole sample and in the two spectra. See the Supplementary Materials for the results of the correlation analyses between accuracy (number of items named correctly by each patient) and the metabolism in the 11 ROIs.

**Table 5:** Results of the correlation analyses between the ROIs metabolism and the weights of the 4 components, i.e., word form, visual, lexical and semantic in the whole sample and in the AD and FTD spectra

	**Whole sample**				**AD spectrum**				**FTD spectrum**			
	word form	visual	Lexical	semantic	word form	visual	lexical	semantic	word form	Visual	lexical	semantic
L middle occipital gyrus, r (*p*-value)	-.023 (.766)	.013 (.861)	.120 (.113)	-.048 (.528)	-.069 (.539)	-.098 (.380)	-.011 (.919)	-.025 (.826)	-.024 (.846)	.087 (.481)	.044 (.722)	.042 (.733)
L pSTG, r (*p*-value)	.182 (.016)	.012 (.874)	-.021 (.785)	-.026 (.730)	.216 (.052)	.020 (.858)	-.165 (.140)	**-.250 (.024)**	.063 (.612)	.042 (.731)	-.041 (.741)	.144 (.242)
L pMTG, r (*p*-value)	**.230 (.002)**	.035 (.650)	-.084 (.270)	-.040 (.598)	.217 (.050)	-.046 (.682)	-.201 (.070)	-.177 (.113)	.158 (.99)	.162 (.187)	-.148 (.229)	.068 (.581)
L pFUS, r (*p*-value)	.156 (.039)	**.223 (.001)**	-.163 (.031)	.050 (.513)	.186 (.094)	-.004 (.968)	-.258 (.019)	-.141 (.206)	.171 (.163)	**.457 (<.001)**	-.162 (.187)	**.321 (.008)**
L middle temporal pole, r (*p*-value)	**.258 (<.001)**	**.207 (.003)**	**-.398 (<.001)**	.063 (.407)	**.424 (<.001)**	.028 (.806)	**-.424 (<.001)**	-.203 (.068)	**.280 (.021)**	**.372 (.001)**	**-.428 (<.001)**	.130 (.290)
L aITG, r (*p*-value)	**.308 (<.001)**	**.255 (<.001)**	**-.413 (<.001)**	.023 (.759)	**.436 (<.001)**	.045 (.688)	**-.459 (<.001)**	**-.291 (.008)**	**.276 (.023)**	**.458 (<.001)**	**-.417 (<.001)**	.167 (.174)
L IFGorb, r (*p*-value)	.108 (.157)	-.002 (.976)	**-.243 (.001)**	.0001 (.997)	.181 (.103)	.001 (.995)	**-.271 (.014)**	-.075 (.505)	.051 (.682)	-.002 (.986)	-.249 (.041)	-.117 (.341)
L IFGtri, r (*p*-value)	.083 (.273)	-.080 (.204)	-.087 (.254)	-.030 (.695)	.148 (.186)	-.115 (.304)	-.153 (.171)	-.054 (.629)	.006 (.961)	-.112 (.365)	-.088 (.478)	-.194 (.112)
L IFGoper, r (*p*-value)	.104 (.171)	-.096 (.204)	-.061 (.421)	-.006 (.939)	.175 (.115)	-.184 (.097)	-.164 (.141)	-.115 (.305)	-.021 (.862)	-.141 (.250)	-.036 (.771)	-.206 (.092)
L supramarginal gyrus, r (*p*-value)	**.193 (.011)**	-.011 (.887)	.080 (.291)	-.046 (.546)	.185 (.096)	-.143 (.200)	-.019 (.868)	-.161 (.149)	.155 (.207)	.062 (.617)	.015 (.902)	.006 (.960)
L inferior parietal cortex, r (*p*-value)	.091 (.230)	-.053 (.487)	.134 (.078)	-.113 (.137)	.023 (.839)	-.066 (.556)	.050 (.652)	-.038 (.735)	.074 (.548)	-.046 (.710)	.027 (.826)	-.224 (.066)

*r* Pearson correlation coefficient, *aITG* anterior inferior temporal gyrus, *IFGtri* inferior frontal gyrus pars triangularis, *IFGoper* inferior frontal gyrus pars opercularis, *IFGorb* inferior frontal gyrus pars orbitalis, *pFUS* posterior fusiform gyrus, *pMTG* posterior middle temporal gyrus, *pSTG* posterior superior temporal gyrus, Significant, Bonferroni-Hochberg corrected results are in bold

In the whole sample and in the FTD spectrum positive correlations emerged between the visual component and metabolism in pFUS, aITG, and temporal pole, indicating that higher metabolism in these areas was associated with the ability to name correctly visually difficult items.

While the semantic component had no significant correlation in the whole sample, it correlated in opposite directions in the AD and FTD spectra. In the AD spectrum, the semantic component was negatively correlated with the metabolism of aITG and pSTG, i.e. higher metabolism in these areas was associated with the ability to correctly name semantically difficult items. In FTD spectrum, the semantic component was positively correlated the metabolism of pFUS, i.e. higher metabolism in this area was associated with correct naming of semantically easier items. To better interpret the divergent correlation directions in the AD and FTD spectra for semantic component, we compared regional metabolism of the ROIs implicated in the results, namely the aITG, pSTG, and pFUS, between the two spectra. While FTD showed higher metabolism in the pSTG (*p* = 0.02) and pFUS (*p* = 0.03) in respect to AD, no significant difference was found in the aITG (*p* = 0.12). This pattern does not match the expected region-specific metabolic differences based on the correlations with the semantic component.

In the whole sample the lexical component showed negative correlations with the metabolism in aITG, temporal pole, and IFGorb, showing that a higher metabolism in these areas was associated with correct naming of lexically difficult items. The same results were reported in the AD spectrum, while in FTD spectrum the correlations were restricted to the aITG and temporal pole.

Positive correlations emerged in the whole sample between the word form component and the brain metabolism in pMTG, aITG, temporal pole, and supramarginal gyrus, i.e. a higher metabolism in these areas was associated with correct naming of items with a difficult word form. In both AD and FTD spectra, the word form component positively correlated with the brain metabolism in aITG and temporal pole.

## Discussion

Although it is acknowledged that picture naming performance is affected by the properties of the pictures and their names [3, 83], the cerebral network on which the variables exert their influence is still not fully understood, specifically in neurodegenerative patients, as none imaging study has been reported on this topic. The present study uniquely combines a large, diverse cohort of neurodegenerative patients, in which naming accuracy is potentially affected by different mechanisms of impairment, with a fine-grained analysis of psycholinguistic and semantic properties, and of metabolic data. Results revealed a distributed neural network that shows both commonalities and specificities in how different stimuli’s properties affected naming accuracy. Furthermore, by analysing patients in the AD and FTD spectra, we show for the first time how distinct pathological and topographic profiles shape the influence of these variables, highlighting potential compensatory plasticity mechanisms.

### Psycholinguistic variables in neurodegenerative diseases (whole sample)

The *visual component* captures the richness of visual information conveyed by the stimuli, namely their visual complexity and the relevance that visual features have on the ‘core’ meaning of the concept itself. Visual information operates at the perceptual level, enabling visual object recognition [2, 108]. We did not find an effect of this component on behavioral performance, aligning with prior behavioral evidence showing that visual information has only a minor [2, 9] or null contribution to picture naming performance [113], but see [37] reporting an effect of visual complexity on picture naming). Accordingly, the effect is suggested to be context-dependent, for example it is present when the objects to be named belong to structurally similar classes [87].

Correctly naming items with a higher visual component score, i.e. more visually difficult, correlated with higher metabolism in pFUS, aITG, and temporal pole. These findings are in line with the proposed role of the aforementioned areas as belonging to the ventral pathway of object recognition, characterized by a caudal-to-rostral gradient of progressive increase in computational complexity [103]. The pFUS has a role in the processing of visual features [51], supporting the differentiation of an object from similar visual or semantic competitors [58, 88], particularly when an object shares many features with the other exemplars of the category [104]. The aITG is specialized for representing the high-level visual-semantic similarity within conceptual categories [16], while the temporal pole has been robustly implied in semantic knowledge [23, 75, 81], representing concepts at the highest level of specificity [11].

The *semantic component* score is measuring the degree of conceptual relevance and semantic distance. The presence of highly relevant conceptual features is helpful to differentiate the target from similar exemplars [19, 20, 95]. Similarly, a higher semantic distance of the concept from the centroid or ‘average exemplar’ of the respective category has been shown to facilitate object identification [38, 118]. In particular, a large overlap of features, i.e. low semantic relevance and closeness to the centroid, has been suggested to hinder picture naming in AD [118]. In our study, however, the semantic component did not impact on behavioral performance. This result aligns with previous evidence showing only a minor or null effect of semantic properties, and may be attributed to the high variability among participants [64] or to differences in severity of the naming impairment and in the type of features considered [20]. At the neural level, significant correlations were found only when the analysis was conducted independently in the two spectra, with opposite directions (i.e. positive and negative correlations, see below), accounting for the absence of results in the entire sample.

The *lexical component* encompasses AoA, frequency, and familiarity, included in the same ‘lexical usage’ component by previous studies [3, 118], in line with the observation that items acquired early in life are usually also frequent and familiar [27]. We found that stimuli with higher scores in the lexical component were more often named correctly. The presence of the effect of lexical variables on picture naming performance is debated, with prior studies either reporting an effect or failing to show it [27, 57, 60, 62, 77, 80, 85]. In this context, studies using updated frequency norms and larger sets of stimuli are more likely to report a frequency effect [2, 100].

At the neural level, we found that correctly naming more lexically difficult items correlated with an increased metabolism of the aITG, temporal pole, and IFGorb. While the first two regions are also correlated with the visual (see above) and the word form (see below) components, the latter is specific for the lexical component. The contribution of lexical properties to naming performance is mainly associated to the lexical retrieval process [56, 89], but other stages of picture naming, including semantic processing, are likely to be involved [27, 60, 77, 115]. In line with the latter view, we found that the metabolism in the anterior temporal lobe is modulated by the lexical properties, which plausibly affect the semantic richness of the stimuli. Different lines of research indeed suggested that early acquired [34], familiar [14], and frequent items [102] are richly interconnected, easily accessible [13], encountered more often and hence characterized by a detailed semantic representation [14]. The IFGorb has been implicated in lexical selection during spoken word production, where it appears to facilitate the suppression of competing lexical entries [32]. The IFGorb may also contribute to solve semantic competition [1] through its functional coupling with temporal regions involved in semantic processing, such as the fusiform gyrus [15].

The *word form component* includes number of letters, number of syllables, and phonological neighborhood. Word length and phonological neighborhood have been consistently reported to be negatively correlated [44], and to cluster together into the same ‘phonological complexity’ component [3]. Our behavioural findings indicate that stimuli with a simple word form, i.e. short and with many phonological neighbours, tend to be named correctly more often, in line with previous literature [26, 60, 72, 85, 112].

At the neural level, correctly naming stimuli with a complex word form was associated with a higher metabolism in supramarginal gyrus, pMTG, aITG, and temporal pole. According to several proposals, word form properties affect the naming stages from phonological encoding onwards [72, 115], with a possible role in modulating lexical-phonological feedback loops, leading to an increasing convergence towards target phonemes [72]. Error-free phonological production in object naming has been linked to the preservation of the dorsal route [98], here represented by the supramarginal gyrus. In picture naming, the supramarginal gyrus shows a role in phonological encoding [84] and hypometabolism in this area is associated to the production of phonological errors in AD patients [54]. The supramarginal gyrus is also involved in processing the phonological distance between words [47] and suggested to enable phonological working memory operating as a storage for phonological information [66]. An interconnection between supramarginal gyrus and pMTG has been already reported in picture naming tasks [36]. In agreement with our finding, the contribution of pMTG to word form properties has been reported in several other studies involving picture naming [52, 74], and hypometabolism in this region correlates with the production of phonological errors in PPA patients [94]. There is however evidence that its role extends to other aspects of language processing, including semantics and syntax [28, 68]. As previously discussed, aITG and temporal pole have a major role in semantic processing. The contribution of these areas to the word form component is debated and may reside on their involvement in phonological processes, such as the production of disyllabic compared to monosyllabic words [40], the elaboration of phonologically relevant information [78], and a role in auditory encoding [49].

### Psycholinguistic variables in the AD and FTD spectra

The neural correlates exhibited by the two spectra for the different components are mainly located in the anterior temporal regions, with additional involvement of the pFUS in FTD patients, and of pSTG and IFGorb in AD patients. The main differences between AD and FTD are related to the visual and semantic components, while the results are almost overlapping for the lexical and word form components. The differences in the correlation patterns we found may in part arise from the relatively distinct topography of neurodegeneration of the two spectra [63], and represent an element of novelty of the present work.

#### AD spectrum

In the AD spectrum only the *visual component* had no significant correlation at the neural level, while the *semantic component* was negatively correlated with the metabolism of the aITG and pSTG. Specifically, naming semantically difficult items was associated with higher metabolism in these areas. Anterior temporal cortices are usually relatively spared in these conditions [24, 96], as also suggested by the hypometabolic pattern in our AD group, and their recruitment is probably required to name semantically difficult stimuli. The involvement of pSTG in the semantic component is less straightforward. A role of the mid-posterior superior temporal gyrus in the retrieval of semantic information has been suggested [93]. Aligning with our findings, pSTG is showed to support lexical retrieval during picture naming in lvPPA patients [73]. It is possible that the more preserved anterior temporal areas trigger the residual functionality of pSTG in the AD spectrum, as spared connections linking anterior and posterior lateral temporal cortex have also been reported in these patients [97].

#### FTD spectrum

In the FTD spectrum, as reported for the whole sample, the *visual component* showed a positive correlation with the pFUS, the left middle anterior temporal pole, and the aITG, with the correct naming of visual complex stimuli associated with increased metabolic activity in these regions. The semantic component positively correlated only with the pFUS, with the correct naming of semantically easier stimuli associated with increased metabolic activity. In FTD, in particular in svPPA and bvFTD, picture naming impairment is usually attributed to a semantic memory disorder due to the involvement of the anterior portions of the temporal lobe [101]. One possible interpretation is that the extraction and elaboration of visual features, supported by the more posterior temporal cortices, such as the pFUS, is relatively spared. This could, in turn, boost the contribution of the anterior areas like the temporal pole and the aITG [114]. In this scenario, spared visual information might drive object identification and support residual semantic resources. Supporting this notion, the hypometabolic pattern observed in the FTD spectrum showed impairment of the temporal pole, while more posterior temporal regions, including the fusiform gyrus, appeared relatively preserved. In the case of the *semantic component* a positive association was found between semantically simpler stimuli and metabolism in pFUS. This result may be interpreted as suggesting that patients attempted to identify the easier stimuli by relying on visual information retained in the preserved occipito-temporal areas. Notably, these posterior occipito-temporal areas did not show hypometabolism in our FTD sample. Previous evidence showed that defective semantic knowledge is compensated by increased perceptual processing in svPPA, with preserved performance in an easy semantic task of categorization involving visual stimuli [10]. This hypothesis aligns with the findings from a previous study by our group [21]. In that study, the presence of semantic errors in FTD patients was associated with increased connectivity between occipital regions and pFUS, which may indicate an increased reliance on visuo-perceptual analysis during picture naming. At the same time, reduced connectivity between the anterior fusiform and the ATL plausibly suggested a failure in mapping perceptual information onto conceptual meaning [21].

Interestingly, the semantic component showed an opposite pattern of correlation in the two spectra. To better interpret these results, we have compared the metabolism of the involved regions, i.e. pFUS, aITG, pSTG, between the AD and FTD spectra. The metabolism in the aITG did not differ between groups, while the FTD exhibited higher metabolism than the AD in both the pSTG and the pFUS. Accordingly, the results cannot be completely accounted by the metabolic baseline. Indeed, the aITG should have correlated in the same direction across both spectra, given the absence of metabolic differences between the AD and FTD groups. Additionally, although both the pSTG and pFUS were more hypometabolic in the AD group compared to the FTD group, only the pSTG showed a significant negative correlation with the semantic component in the AD group. Therefore, the correlations with the semantic component in opposite directions may reflect a combination of the hypometabolic patterns and potential compensatory mechanisms specific to each spectrum.

### Limitations

Although we included different neurodegenerative conditions to provide a comprehensive anatomical coverage of the impaired brain regions, the groups were not equally represented, leading to a numerical imbalance that may have limited the detection of region-specific effects. For example, the PCA group represented only approximately 4% of the patients, thereby limiting the possibility to assess the involvement of the occipital regions, known to be affected in this group.

Moreover, the left hemisphere was well represented by the anatomical profile of the patients in our sample, with PPA representing more the 50% of our sample. The comparative underrepresentation of the right hemisphere may have limited the detection of effects related to the psycholinguistic variables under investigation. As a result, we decided to focus our analyses exclusively on left-hemispheric regions. Nonetheless, we acknowledge the likely contribution of right-hemispheric structures and emphasize the need for their systematic investigation in future research.

## Conclusions

In conclusion, we reported that a simple word form and lexical structure were associated with better naming performance in neurodegenerative patients, aligning with previous findings, and offering noteworthy insights for the construction and selection of suitable naming test to be used in diagnostic and rehabilitation settings. The psycholinguistic properties, that we assessed only in a left-lateralized brain network, impacted on the temporal lobe, ranging from the posterior to the most anterior portions, as well as the IFGorb and the supramarginal gyrus, previously implied in naming tasks in both healthy and neurological populations [21, 48, 74, 84, 115, 117]. The inclusion of a large sample of neurodegenerative patients, as well as the investigation of different psycholinguistic and semantic properties represent a key novelty of the current study, that allowed us to highlight a distributed neural network that shows both commonalities and specificities in how stimulus and word properties influence naming accuracy. Furthermore, the inclusion of patients from both the AD and FTD spectra allowed us, for the first time, to examine how distinct brain topographies and underlying pathological mechanisms influence the neural correlates of psycholinguistic variables. Based on these findings, the brain network described above appears capable of engaging in compensatory plastic changes. Such adaptations may help sustain residual naming performance despite the progression of neurodegeneration, particularly in regions that remain relatively spared, hence providing a neural substrate to support cognitive intervention for naming in neurodegenerative diseases. Overall, our view aligns with the models of spoken word production which assume a cascading of activation and interactivity between processing levels, going beyond strictly serial models [8]. Indeed, although the same regions may be associated with different components, their roles are based on the specific connections they receive. In this context, while all component processes may engage the same regions, one process could be more dependent on the activity of some regions (and the connections between them) than others [29]. Graded specificities could be in fact detected, with pFUS supporting both visual and semantic features, and inferior frontal and posterior temporal/parietal cortices representing an interface between lexico-semantic and phonological properties. The anterior temporal lobe appears to contribute to all stages of the picture naming process, with a possible role connecting lexical and phonological units, extending to phonological encoding [54].

## Supplementary Information

Supplementary Material 1

## Data Availability

The datasets used and/or analyzed during the current study are available from the corresponding author after a reasonable request.

## Abbreviations

AAL: Automated Anatomical Labelling
AD: Alzheimer’s Disease
aITG: Anterior inferior temporal gyrus
AoA: Age of Acquisition
bvFTD: Behavioral variant of frontotemporal dementia
CBS: Corticobasal syndrome
DLB: Dementia with Lewy bodies
EANM: European Association of Nuclear Medicine
FAM: Familiarity
FREQ: Frequency
FTD: Frontotemporal Dementia
IFGoper: Inferior frontal gyrus opercularis
IFGorb: Inferior frontal gyrus pars orbitalis
IFGtri: Inferior frontal gyrus, pars triangularis
KMO: Kaiser–Meyer–Olkin
lvPPA: Logopenic variant of primary progressive aphasia
MMSE: Mini-Mental State Examination
