# Supplementary material for: Mapping the neural correlates of the effect of psycholinguistic variables on picture naming performance: a FDG-PET study across neurodegenerative diseases
Source: Alzheimers Res Ther. 2025 Dec 20;17:272. doi: 10.1186/s13195-025-01936-y (PMC12751676; doi:10.1186/s13195-025-01936-y)
Supplement: Supplementary file 1 — Supplementary Material 1 [file 13195_2025_1936_MOESM1_ESM.docx]

**Supplementary materials**

**Table 1.** Studies investigating the neural correlates of psycholinguistic variables in different populations

|  | **Alyahya et al., 2020** | **Graves et al., 2007** | **Wilson et al., 2009** | **Ellis et al., 2006** | **Urooj et al., 2014** | **Miozzo et. al., 2015** | **Gertel et al., 2020** | **Campo et al., 2016** | **Okada et al., 2003** | **Xu et al., 2020** |
| --- | --- | --- | --- | --- | --- | --- | --- | --- | --- | --- |
| population | Ps | H | H | H | H | H | H | El, H | H | H |
| length | phonemes | Syllables | Phonemes |  |  | phonemes |  | letters | syllables |  |
| phonological neighbourhood density |  |  |  |  |  |  |  |  |  |  |
| frequency |  |  |  |  |  |  |  |  |  |  |
| familiarity |  |  |  |  |  |  |  |  |  |  |
| age of acquisition |  |  |  |  |  |  |  |  |  |  |
| semantic diversity |  |  |  |  |  |  |  |  |  |  |
| imageability |  |  |  |  |  |  |  |  |  |  |
| visual complexity |  |  |  |  |  |  |  |  |  |  |
| distinguishing features |  |  |  |  |  |  |  |  |  |  |
| encyclopedic features |  |  |  |  |  |  |  |  |  |  |
| ways to interact with objects |  |  |  |  |  |  |  |  |  |  |
| ease to mime actions in response to objects |  |  |  |  |  |  |  |  |  |  |

H = healthy subjects; Ps = post-stroke patients; El = patients with epileptogenic lesions. The variables investigated are in grey.

**Maps of hypometabolism in the two spectra**

We produced maps of hypometabolism to show the most affected regions in the AD and FTD spectra. The pre-processed single-subject images (see main text for details) were entered in the analysis. We used a two samples t-test to compare the patients’ group of interest against the dataset of healthy controls. Figure 1 and Table 2 below show the statistically significant results at a significance level p < 0.05 after applying a familywise error (FWE) correction for multiple comparisons, for clusters of at least 200 voxels. In the FTD spectrum the main areas of hypometabolism are located in the left hemisphere and include the insular cortex, the temporal pole, extending to the planum polare, paracingulate cortex, as well as the inferior and the middle frontal gyri. Hypometabolism further includes the left caudate, subcallosal cortex and the bilateral thalamus, the left angular gyrus, the left medial frontal cortex. In the AD spectrum the pattern of hypometabolism extend more bilaterally. The main clusters are located in the bilateral lateral occipital cortex, extending to the left angular and supramarginal gyri, the posterior section of middle/inferior temporal cortex, and the bilateral caudate. Other clusters encompass the bilateral precuneus and the left middle frontal gyrus, the left cingulate cortex and the left inferior frontal gyrus.


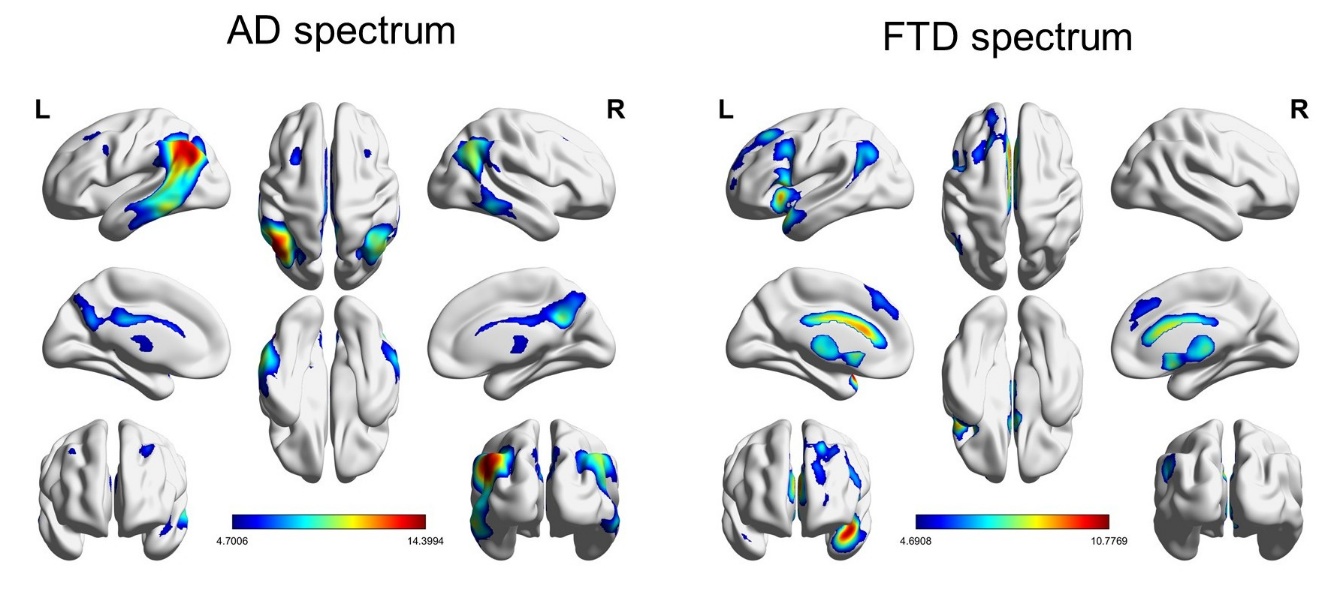


**Figure 1.** Anatomical rendering of the maps of hypometabolism for Alzheimer’s Disease (AD) spectrum and the Fronto-Temporal Dementia (FTD) spectrum. Images represent the statistical parametric mapping t map obtained from a two samples t-test comparing the patients’ group against the dataset of healthy controls.

**Table 2.** Area of hypometabolism in the Alzheimer’s Disease (AD) spectrum and the Fronto-Temporal Dementia (FTD) spectrum.

| **Cluster number** | **Number of voxels** | **MNI coordinates of the cluster** | **Local maxima within the cluster (label assigned with Anatomy Toolbox)** |
| --- | --- | --- | --- |
| ***AD spectrum*** | | | |
| cluster 1 | 12709 | -48, -68, 32 | L lateral occipital/inferior parietal lobule; L angular gyrus; L posterior middle temporal gyrus; L posterior inferior temporal gyrus; L/R caudate; L supramarginal gyrus |
| cluster 2 | 5508 | 46, -62, 30 | R lateral occipital cortex |
| cluster 3 | 5154 | 0, -72, 36 | R/L Precunues; L cingulate cortex |
| cluster 4 | 705 | -32, 12, 46 | L middle frontal gyrus; L inferior frontal gyrus |
| ***FTD spectrum*** | | | |
| cluster 1 | 12019 | -40, 18, -6 | L insular cortex; L temporal pole; L planum polare; L paracingulate cortex; L inferior frontal gyrus; L middle frontal gyrus |
| cluster 2 | 3711 | -10, 14, 10 | L caudate; R subcallosal cortex; R/L thalamus |
| cluster 3 | 1224 | -50, -52, 22 | L angular gyrus |
| cluster 4 | 243 | -2, 32, -32 | L medial frontal cortex |

**Table 3.** List of the 11 Regions of Interest (ROIs) with the respective MNI coordinates taken from AAL atlas

| Label | X | Y | Z |
| --- | --- | --- | --- |
| L middle occipital gyrus | -32.39 | -80.73 | 16.11 |
| L superior temporal gyrus (posterior section) (pSTG)* | -53.16 | -20.68 | 7.13 |
| L middle temporal gyrus (posterior section) (pMTG)* | -55.52 | -33.8 | -2.2 |
| L fusiform gyrus (posterior section) (pFUS)* | -31.16 | -40.3 | -20.23 |
| L middle temporal pole | -36.32 | 14.59 | -34.08 |
| L inferior temporal gyrus (anterior section) (aITG)* | -49.77 | -28.05 | -23.17 |
| L inferior frontal gyrus (pars orbitalis) (IFGorb) | -35.98 | 30.71 | -12.11 |
| L inferior frontal gyrus (pars triangularis) (IFGtri) | -45.58 | 29.91 | 13.99 |
| L inferior frontal gyrus (pars opercularis) (IFGoper) | -48.43 | 12.73 | 19.02 |
| L supramarginal gyrus | -57.61 | -31.5 | 34.48 |
| L inferior parietal cortex | -42.8 | -45.82 | 46.74 |

L = left; * = regions divided into anterior and posterior sections, see text for details.

**Correlations between psycholinguistic variables**

Significant negative correlations emerged between AoA and, respectively, FREQ (r = - 0.51), FAM (r = -0.44), and phonological neighbourhood (r = - 0.43). Positive correlations were found between visual complexity and visual relevance (r = 0.66). The number of syllable and letters positively correlated (r = 0.79), and both were negatively correlated with phonological neighbourhood (r = -0.58 and r = -0.63, respectively. See Figure 2 for the correlation matrix. No correlations exceeded |0.8|.


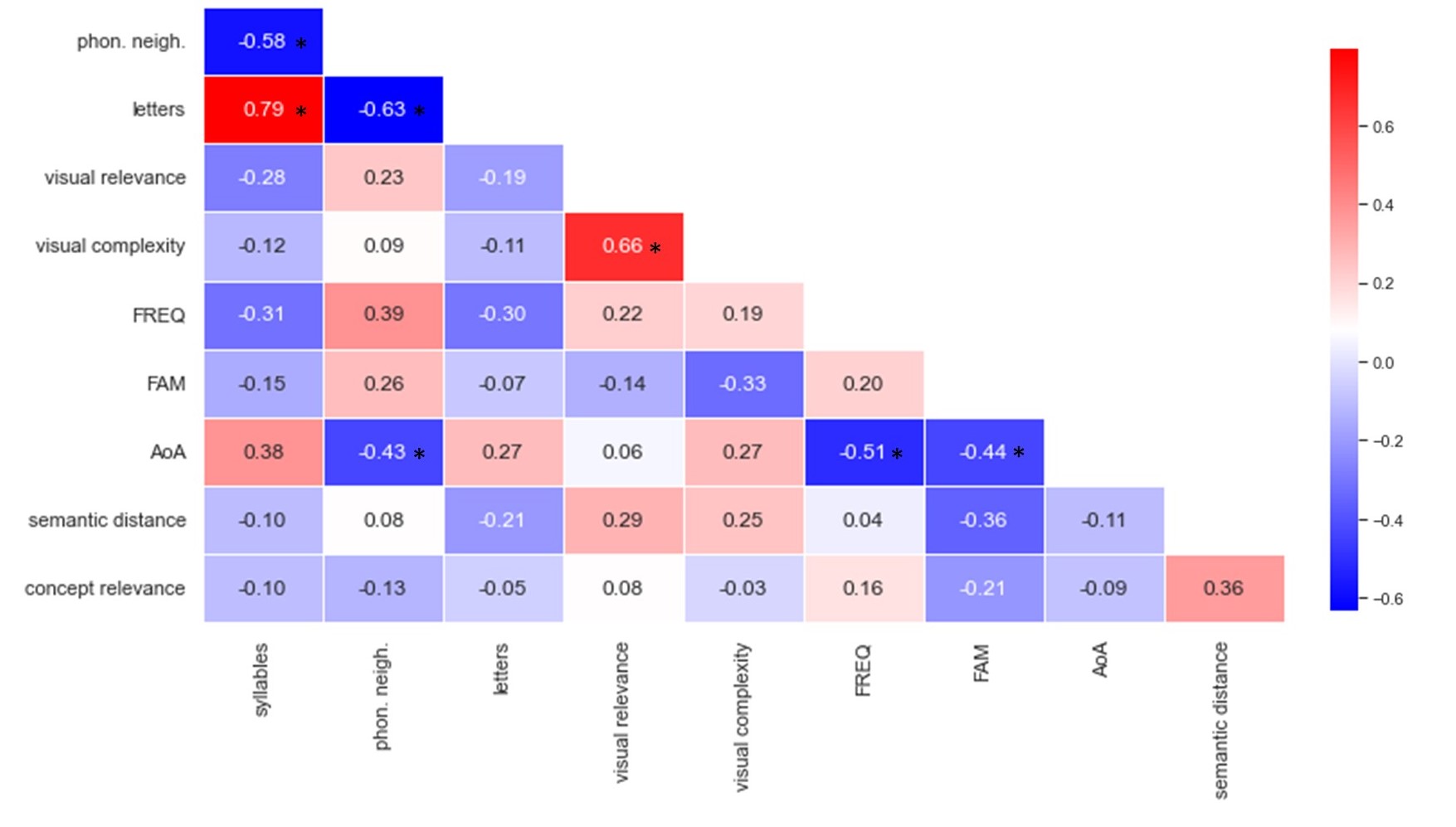


**Figure 2.** Correlation matrix reporting the Pearson correlation coefficients, * = significant, Bonferroni- Hochberg corrected. Red and blue tiles indicate positive and negative correlations, respectively, with the density of the colours showing the strength of the correlations; AoA = age of acquisition, FAM = familiarity, FREQ = frequency, phon. neigh = phonological neighbourhood.

**Table 4.** Results of the correlation analyses between the ROIs metabolism and the accuracy (namely the proportion of correctly named items) in the whole sample and in the AD and FTD spectra.

|  | **Whole sample** | **AD spectrum** | **FTD spectrum** |
| --- | --- | --- | --- |
| L middle occipital gyrus, r (p-value) | -.144 (.058) | .041 (.713) | -.010 (.936) |
| L pSTG, r (p-value) | .133 (.078) | .245 (.026) | .181 (.139) |
| L pMTG, r (p-value) | .218 (.004) | .307 (.005) | .346 (.004) |
| L pFUS, r (p-value) | .245 (.001) | .310 (.005) | .373 (.002) |
| L middle temporal pole, r (p-value) | .556 (<.001) | .483 (<.001) | .627 (<.001) |
| L aITG, r (p-value) | .601 (<.001) | .556 (<.001) | .660 (<.001) |
| L IFGorb, r (p-value) | .303 (<.001) | .246 (.026) | .274 (.024) |
| L IFGtri, r (p-value) | .151 (.046) | .151 (.174) | .099 (.420) |
| L IFGoper, r (p-value) | .115 (.131) | .173 (.121) | .013 (.919) |
| L supramarginal gyrus, r (p-value) | .052 (.496) | .140 (.211) | .176 (.152) |
| L inferior parietal cortex, r (p-value) | -.098 (.198) | -.011 (.919) | .015 (.904) |

r = Pearson correlation coefficient; aITG = anterior inferior temporal gyrus; IFGtri = inferior frontal gyrus pars triangularis; IFGoper = inferior frontal gyrus pars opercularis; IFGorb = inferior frontal gyrus pars orbitalis; pFUS = posterior fusiform gyrus; pMTG = posterior middle temporal gyrus; pSTG = posterior superior temporal gyrus; Significant, Bonferroni-Hochberg corrected results are in grey.

**Supplementary references**

Alyahya, R. S., Halai, A. D., Conroy, P., & Ralph, M. A. L. (2020). Mapping psycholinguistic features to the neuropsychological and lesion profiles in aphasia. Cortex, 124, 260-273.

Campo, P., Poch, C., Toledano, R., Igoa, J. M., Belinchón, M., García-Morales, I., & Gil-Nagel, A. (2016). Visual object naming in patients with small lesions centered at the left temporopolar region. Brain Structure and Function, 221, 473-485.

Ellis, A. W., Burani, C., Izura, C., Bromiley, A., & Venneri, A. (2006). Traces of vocabulary acquisition in the brain: Evidence from covert object naming. NeuroImage, 33(3), 958-968.

Gertel, V. H., Karimi, H., Dennis, N. A., Neely, K. A., & Diaz, M. T. (2020). Lexical frequency affects functional activation and accuracy in picture naming among older and younger adults. Psychology and Aging, 35(4), 536.

Graves, W. W., Grabowski, T. J., Mehta, S., & Gordon, J. K. (2007). A neural signature of phonological access: distinguishing the effects of word frequency from familiarity and length in overt picture naming. Journal of cognitive neuroscience, 19(4), 617-631.

Miozzo, M., Pulvermüller, F., & Hauk, O. (2015). Early parallel activation of semantics and phonology in picture naming: Evidence from a multiple linear regression MEG study. Cerebral Cortex, 25(10), 3343-3355.

Okada, K., Smith, K. R., Humphries, C., & Hickok, G. (2003). Word length modulates neural activity in auditory cortex during covert object naming. Neuroreport, 14(18), 2323-2326.

Urooj, U., Cornelissen, P. L., Simpson, M. I., Wheat, K. L., Woods, W., Barca, L., & Ellis, A. W. (2014). Interactions between visual and semantic processing during object recognition revealed by modulatory effects of age of acquisition. NeuroImage, 87, 252-264.

Wilson, S. M., Isenberg, A. L., & Hickok, G. (2009). Neural correlates of word production stages delineated by parametric modulation of psycholinguistic variables. Human brain mapping, 30(11), 3596-3608.

Xu, Z., Shen, B., Taji, W., Sun, P., & Naya, Y. (2020). Convergence of distinct functional networks supporting naming and semantic recognition in the left inferior frontal gyrus. Human Brain Mapping, 41(9), 2389-2405.
